# How to Motivate Employees for Sustained Innovation Behavior in Job Stressors? A Cross-Level Analysis of Organizational Innovation Climate

**DOI:** 10.3390/ijerph16234608

**Published:** 2019-11-20

**Authors:** Pei-Xu He, Tung-Ju Wu, Hong-Dan Zhao, Yang Yang

**Affiliations:** 1 Oriental Enterprise Management Research Center & Research Center of Business Management, School of Business Administration, Huaqiao University, Quanzhou 362021, China; hepeixu@hqu.edu.cn; 2 School of Management, Harbin Institute of Technology (HIT), Harbin 150001, China; tjwu@hit.edu.cn; 3 School of Management, Shanghai University, Shanghai 200444, China; jimmyzhaoxin@shu.edu.cn

**Keywords:** challenge stressors, hindrance stressors, organizational innovation climate, sustained innovation behavior, creative self-efficacy

## Abstract

The starting point of organizational innovation is employees’ creative thinking and innovation behaviors at work. In addition to personality and innovation willingness, innovation behavior depends on the level of support available in an organizational environment. The data used in this study were collected from 74 R&D teams (418 employee participants) in technology companies in Taiwan, and a multi-level analysis was conducted to investigate the relationships among job stressors, creative self-efficacy, and employees’ sustained innovation behavior, as well as the role of the organizational innovation climate between creative self-efficacy and employees’ innovation behavior. The research findings revealed significant positive relationships between challenge stressors and employees’ sustained innovation behavior, as well as significant negative relationships between hindrance stressors and employees’ sustained innovation behavior, mediation effects of creative self-efficacy on job stressors and employees’ sustained innovation behavior, and moderation effects of the organizational innovation climate on employees’ creative self-efficacy and sustained innovation behavior. An enterprise could place some working-related stress on employees and create a rich internal innovative climate to induce innovation behavior in its members.

## 1. Introduction

As suggested by the saying “innovate or die”, innovation has become the major source of corporate competitive advantage. The starting point of innovation lies in members presenting innovation behavior at work, including the application of creativity, identifying problems, the use of opportunities, positive development of creativity, and the implementation of creativity to promote new products and new services and even open an innovation market [1]. Anderson et al. [2] reviewed the research on the creativity and innovation of modern organizations and stated that the relevant research can be divided into two categories: Creative thinking (creativity) and creative implementation (innovation behavior). While organizational innovation generally involves the creative thinking of individuals or teams, creative thinking is not a prerequisite for organizational innovation. Members can engage in innovative activities through new practices from other industries or foreign organizations [3]. In other words, innovation behavior includes two general stages: Creative thinking and creative implementation. The organizational creativity research focuses on the members’ creative cognition processes; organizational innovation behavior research is concerned with the interpersonal and socialization of innovation behavior. In this case, either inducing members’ development of creativity or encouraging the implementation of creativity are issues that concern organizational innovation researchers [2,3,4,5]. According to the job demand-resource model (JDR), job demands and job resources may have different effects on individuals’ job attitudes and behavioral performance. Job demands mean that the organization requires the individual to pay more attention at work. However, if the job requires more than workers can provide, it is likely to cause stress.

The workplace is full of various stressors. Stress might provide an opportunity or challenge for an individual, but it is also a threat and source of frustration. Determining how to handle such stressors, therefore, has become an important issue. Stressors, as factors in the stress response, might be positive or negative. Positive stress refers to opportunities being offered for acquiring expected objects; negative stress imposes restrictions or requirements [6]. Working pressure is an important factor in individuals’ working attitudes and their physical and mental health [7]; sustained working pressure can have adverse effects on individuals’ psychological and behavior performance [6]. Cavanaugh et al. [8] discovered that working pressure could produce positive and negative psychological feelings. Therefore, job stressors are divided into challenge stressors and hindrance stressors. Challenge stressors are generally regarded as positive stress factors on an individual, where work requirements are regarded as development opportunities to enhance self-development and achieve goals, such as a suitable workload, the pressure of the supervisor during task completion, or the urgency of the deadline. While challenge stressors can induce positive emotions, enhance work motivation and performance, and promote job satisfaction, they can reduce the quality of work and personal well-being [7]. Hindrance job stressors refer to an individual having problems in achieving their goal and work requirements, receiving limited work development, not being able to fulfill their potential, and being hindered in terms of motivation and expectations of continuous progress. For example, conflicts between colleagues, informal political tactics operations, or unclear job responsibilities. Many studies have stressed the negative effects of hindrance job stressors on individual work effectiveness and attitudes [9,10]. The effects of these two different stressors on employees’ innovation behavior were the motivation for this study.

Innovation is a complicated and high-risk behavior that involves many uncertainties. Employees often encounter various problems and risks that produce unclear results when engaging in innovation behaviors regarding creative thinking and problem-solving. Amabile [3] argued that creativity is not only a personal phenomenon, but the organizational context is also an important factor that influences creativity. Therefore, the core of individual creativity should include personal and organizational factors. For this reason, employees interpret organizational support for innovation according to various organizational management practices to determine the presence of creativity and innovation behavior [2,4,5]. The climate formed by various factors in the work environment is called the organizational innovation climate [11,12]. Amabile [3] defined the organizational innovation climate as a perceptual description of the organization’s work environment, including whether the organization is incentivized to innovate, the resources provided by the organization, and the level of innovation in management practices. Many high-tech companies, e.g., Apple, Google, and Facebook, have created comfortable organizational/working climates to encourage employee innovation motivation and enthusiasm. According to the social learning theory, the innovation behavior of organizational members is generated through the self-recognition process [13,14,15]. Creative self-efficacy refers to an individual’s belief and self-confidence that they can achieve specific innovation tasks [16,17,18,19]. In this case, employees have to perceive that the management practices of the organization support innovation and believe in their own ability to achieve innovation tasks. This self-confidence or creative self-efficacy to complete innovation tasks is the primary factor in driving employees to do their best to fulfill innovation tasks [4]. In this study, we also want to examine roles within the innovation climate and creative self-efficacy in employees’ innovation behavior.

The high-tech industry is an economic mainstream in the 21st century. Continuous innovation is required to satisfy consumers’ preferences. To manage continuous innovation behavior, most high-tech organizations undergo organizational-change-related measures to promote corporate competitiveness. As a result, high-tech enterprises must ensure that their employees, when undertaking organization changes, calmly face possible working pressures to ensure sustained organizational performance. As engineers are the core human resources of a high-tech organization, they must be able to effectively manage working pressure and continuously provide a positive output.

In this study, the JDR model is applied to the engineers of high-tech enterprises, who were the participants here, to examine the relationship between challenging job stressors, hindrance job stressors, and employees’ innovation behavior, as well as to clarify the correlations of positive and negative effects between job stressors and innovation behavior through the mediation effect of creative self-efficacy and the cross-level moderation effect of the innovation climate. We expect that the research findings will provide employees with information to understand stress sources, help reduce the impacts of stressors on innovation behavior, and adjust their working attitudes as well as those of organizations by providing references and suggestions for human resource management workers.

### 1.1. Job Stressors, Creative Self-Efficacy, and Sustained Innovation Behavior

According to the evaluation models in the transactional theory of stress, stressors are divided into challenge evaluation stressors and hindrance threat evaluation stressors, which provides the research context for challenge and hindrance stressors [8]. An individual who is able to satisfy various work requirements in a fiercely competitive work field bears a high workload and copes with various problems in the workplace [20]. Combining the perspective of expectancy theory and the cognitive appraisal of stress, individuals consider their own internal and external conditions after regarding stressors as the primary assessment of challenges or threats. Then, they generate a positive secondary appraisal with a successful response or a negative secondary appraisal with failure and helplessness. Consequently, challenge and hindrance stressors can, respectively, induce positive and negative working attitudes and further affect the behavior performance [9,21]. Cavanaugh et al. [8] discovered that an individual who thinks that they are able to handle stressors experiences stress as an opportunity for growth; conversely, some stressors, when regarded as uncontrollable or impossible to handle, hinder an individual from seeking development. Wallace et al. [22] stated that challenge stressors induce individual desire and self-confidence, generating positive problem-solving and handling methods (e.g., working hard and positively dealing with stressors), whereas hindrance stress can result in negative emotions, leading to the use of emotional tactics to manage external threats or anxiety (such as escape, revenge, and distraction).

Hessels et al. [23] conducted an integrated analysis of the results of job stressors and identified three variables related to stress: Physical health, general psychological well-being, and individual job performance. Amabile [2] discovered that moderate working pressure generates a work challenge, enhancing members’ creativity, whereas excessive time pressures restrain members’ creativity. Previous research reported positive relationships between challenge stressors and self-efficacy, job satisfaction, and organizational affective commitment, but negative relationships between hindrance stressors and self-efficacy, job satisfaction, and organizational affective commitment [9,10,24]. In other words, an individual, in the challenge-hindrance model, precedes the cognitive appraisal of stressors and makes classifications. Challenge stress leads the individual to experience a sense of accomplishment, producing positive effects on work effectiveness and attitudes, whereas hindrance stress does not lead to maximizing the individual’s potential, but results in negative effects on work effectiveness and attitude. In this study, we consider that an individual, when involved in stressful challenge tasks, is willing to be involved in, and is devoted to, the organization due to the sense of accomplishment and personal honor experienced, which effectively enhances their efficiency and productivity in the organization. Given stressful hindrance tasks, the powerless feeling of work is enhanced, which reduces the passion and expectations of the original work tasks, and eventually results in negative behavior toward the organization. In sum, we hypothesize the following:

**Hypothesis** **1a:** *Challenge stress has a significant positive relationship with employees’ sustained innovation behavior.*

**Hypothesis** **1b:** *Hindrance stress has a significant negative relationship with employees’ sustained innovation behavior.*

According to the expectancy theory, job stressors after an individual assessment become the internal and external motivational factors for subsequent behaviors. The concept of creative self-efficacy originated from the self-efficacy theory, as proposed by Bandura [25], who stated that self-efficacy influences individual motivation and cognitive resources, further affecting behavior and job performance. An individual with higher self-efficacy has a higher probability of achieving work tasks at a high performance level [26,27]. Orth and Volmer [18] discovered that creative self-efficacy is different from overall work effectiveness and is the key factor in members’ creativity performance. An individual’s previous success, vicarious experiences, persuasion from others, and physiological and emotional states are the major sources of self-efficacy [16,17]. The individual factors of cognition, situation, and behavior interact. An individual who perceives the resources, experience, and received situation information as being beneficial for task achievement can have higher levels of self-efficacy. In other words, self-efficacy, as a dynamic idea, can be accumulated with individual experience (e.g., professional knowledge and success experience) and can increase with the perception in the organization (e.g., supervisor support or resources) [19,26]. Accordingly, an individual thinks that overcoming a stressor with self-effort increases their creative self-efficacy when they perceive work stress as a challenge. Conversely, an individual can easily feel powerless in their job due to the feeling that self-effort cannot overcome such stress; creative self-efficacy, therefore, decreases when work stress is perceived as a hindrance. In sum, we posit the following:

**Hypothesis** **2a:** *Challenge stress has a significantly positive relationship with employees’ creative self-efficacy.*

**Hypothesis** **2b:** *Hindrance stress has a significant negative relationship with employees’ creative self-efficacy.*

**Hypothesis** **3a:** *Employees’ creative self-efficacy has mediation effects on challenge stress and innovation behavior.*

**Hypothesis** **3b:** *Employees’ creative self-efficacy has mediation effects on hindrance stress and innovation behavior.*

### 1.2. Cross-Level Moderating Role of the Organizational Innovation Climate

Organizational members’ innovation behavior mostly depends on their beliefs in organizational support, self-ability, and morale [13,24]. For example, curiosity and discontent enhance innovation behavior, whereas anxiety limits innovation behavior. The belief in the ability of members refers to creative self-efficacy. Studies illustrated the positive correlation between members’ creative self-efficacy and innovation performance. Creative self-efficacy can explain members’ innovation performance better than the overall work effectiveness [28,29,30]. Anderson et al. [2] further confirmed that an increase in members’ creative self-efficacy enhances their innovation performance.

**Figure 1:**
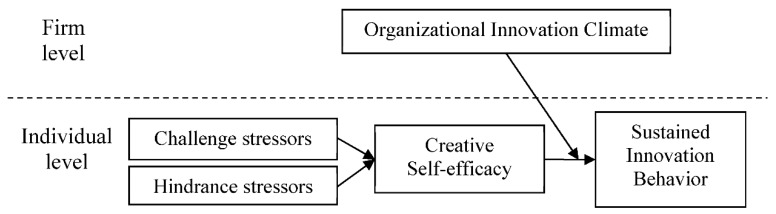
The framework of the effect of job stressors on the relationship between creative self-efficacy, the organizational innovation climate, and sustained innovation behavior.

Amabile [2] emphasized that the organizational innovation climate is the critical factor in the creativity motivation of organizational members, and creativity motivation is a factor in the creativity of members. Shanker et al. [12] indicated the organizational innovation climate as being a key factor, which is mostly emphasized by researchers regarding members’ creativity and innovation as a critical factor in organizational performance [31]. From the social information processing perspective, regardless of an individual being aware of the effect of the social environment, the codes of conduct and information in the social environment context affect individual cognition and the behavior model through personal perception, experience, and evaluation. In this case, an employee, when agreeing with the environment, gains motivation, attitudes, and behaviors toward the regulations and expectations of the environment [32,33]. In this study, we hypothesized that a positive relationship exists between the organizational innovation climate and members’ creativity and innovation performance. When an organization experiences considerable environmental change and is highly competitive, the organizational innovation climate can enhance the employees’ innovation behavior (with larger effects). As a result, we inferred that the organizational innovation climate has significant positive moderation effects on employees’ creative self-efficacy and innovation behavior (Hypothesis 4, H4). The framework of this study is shown in Figure 1.

## 2. Materials and Methods

### 2.1. Participants and Procedures

Research and development (R&D) refers to the activities undertaken by companies to innovate and introduce new products and services. It is often the first stage in the development process. The goal is typically to bring new products and services to the market and add to the company’s bottom line. Considering the R&D teams of technology companies in Taiwan as the research participants, working pressure in technology companies is caused by R&D and innovation, and R&D personnel should present the same innovation behavior when they face different stressors. To reduce the common method variance (CMV) error [34], the supervisor-subordinate pair was used for data collection. Since supervisors of R&D teams had a high level of control and understanding of the R&D personnel’s task performance, supervisors evaluated the innovation behavior. Subordinates responded to items related to job stressors, creative self-efficacy, and the organizational innovation climate.

The questionnaire was administered to 74 R&D teams (including 62 technology companies, 74 supervisors, and 418 R&D employees). The participants were distributed among the following branches: Technology manufacturing (47.7%), the information network industry (28.6%), and telecommunication services (23.7%). The average number in a team was nine, and the average number of respondents in each team was three–six persons (mean: 5 persons). Among the R&D employees, 93.6% were male, the average age was 34.7 years (SD = 5.33), the average experience in the service industry was 6.8 years (SD = 4.84), and the educational background was above graduate school. Of the supervisors, 98.7% were male, the average age was 41.3 years (SD = 7.46), the average experience in the service industry was 11.54 years (SD = 3.19), and the educational background was above graduate school.

### 2.2. Measures

Job Stressors. Referring to Cavanaugh et al. [8], we divided working pressure into challenge and hindrance when developing the scale, which included 11 items. Responses were collected using a Likert scale with 1 indicating “never” and 5 indicating “very often”. Higher scores denoted a higher level of perceived stress. Challenge job stressors included six items, for example, “the number of projects and assignments I have”. The Cronbach’s α in the present study was 0.91. Hindrance job stressors included five items, for example, “the amount of red tape I need to go through to get my job done”. The Cronbach’s α for this section in the present study was 0.88.

Creative Self-Efficacy. The scale developed by Tierney and Farmer [19] allowed employees to evaluate their confidence when engaging in specific innovation tasks. The scale contained three items, for example, “I believe that I have the ability to solve problems in my work with creativity”. The participants’ responses were collected using a Likert scale in which 1 indicated “never” and 5 indicated “very often”. The Cronbach’s α for this scale was 0.92.

Organizational Innovation Climate. This measure was based on the organizational climate model developed by Ekvall [35] and consisted of the following sub-scales: (1) Challenge (8 items), (2) freedom (6 items), (3) trust (3 items), (4) the idea of time (6 items), (5) playfulness (6 items), (6) conflict (6 items), (7) the idea of support (5 items), (8) debate (6 items), and (9) risk-taking (4 items). There were 50 items in total, covering the nine dimensions of the Situational Outlook Questionnaire (SOQ). Employees were asked to indicate the perceived climate for innovation in their organizations according to a five-point Likert scale. Each item was scored from 1 as “never” to 5 as “very often”. Using the second-order single-factor model for the CFA (Confirmatory Factor Analysis, CFA), the overall fit indices (χ^2^ = 221.43, CFI = 0.98, RMSEA = 0.06, NFI = 0.97, AGFI = 0.92, and GFI = 0.95) appeared better than when the first-order nine-factor model was used (χ^2^ = 835.72, CFI = 0.89, RMSEA = 0.09, NFI = 0.93, AGFI = 0.85, and GFI = 0.88). The Cronbach’s α for this scale was 0.91.

Sustained Innovation Behavior. We adopted the innovation behavior scale developed by Lu et al. [13]. In this study, our research object was R&D employees of technology companies. R&D’s goal is to continuously develop innovative performance behaviors for the organization; therefore, this part was answered by the direct supervisor of the employee to determine whether the employee had recently demonstrated sustained innovation. The scale contained six items, for example, “At work, they apply new technologies, procedures, or methods”. This part was answered by supervisors, and responses were collected using a Likert scale, in which 1 represented “never” and 5 represented “very often”. The Cronbach’s α for this scale was 0.95.

Control Variables. Previous research discovered that different sexes present remarkable differences in creativity tests or creativity task-related performance measures [13]. Sex was, therefore, included at the individual level in this study. We found significant differences related to an individual’s age, experience, educational background, and innovation ability [12,15]. For this reason, age and service experience were included at the individual level for the control.

### 2.3. Data Analysis

This study involved employees and organizations, and the multilevel model (MLM) was applied to test the hypotheses [36]. Grand-mean centering was used to manage the MLM centralization problem. Grand-mean centering was used to control the effect of variables at the individual level and enhance the effects of variables at the team level to highlight the MLM test results as well as to decrease the multicollinearity by reducing the correlation between intercept and slope at the team level.

## 3. Results

Table 1 illustrates the mean scores, standard deviations, and correlations among the study variables. At the employee level, R&D personnel age and experience (*r* = 0.33, *p* < 0.01) were found to have a significant positive relationship with challenge job stressors (*r* = 0.18, *p* < 0.05), but significant negative relationships with hindrance job stressors (*r* = −0.21, *p* < 0.05), creative self-efficacy (*r* = −0.19, *p* < 0.05), and sustained innovation behavior (*r* = −0.25, *p* < 0.01). Experience was significantly negatively related to creative self-efficacy (*r* = −0.18, *p* < 0.05). Challenge job stressors showed significant positive relations with hindrance job stressors (*r* = 0.31, *p* < 0.01), creative self-efficacy (*r* = 0.25, *p* < 0.01), and sustained innovation behavior (*r* = 0.31, *p* < 0.01). Hindrance job stressors showed significant negative relationships with creative self-efficacy (*r* = −0.23, *p* < 0.01) and sustained innovation behavior (*r* = −0.28, *p* < 0.01). Creative self-efficacy was found to have a significant positive relationship with sustained innovation behavior (*r* = 0.35, *p* < 0.01). At the unit level, supervisors’ age and experience were found to be significantly positively related (*r* = 0.22, *p* < 0.05).

The collected valid samples were regarded as the unit level variables for the measurement in this study, including 74 R&D units and 418 R&D employees. The average number of employees was 5.65, with the lowest number of employees being three and the highest being six. The inter-consistency of the organizational innovation climate was further evaluated, including Rwg, ICC(1), and ICC(2). The results revealed that the mean Rwg was 0.86, ranging from 0.710 to 0.990; the mean ICC(1) was 0.177; and the mean ICC(2) was 0.622. The between-group variance was also significant (F = 3.32, *p* < 0.001). As shown in Table 2, the five-factor model significantly outperformed the single-factor (Δχ^2^ = 2986.33, *p* < 0.001) and 13-factor (Δχ^2^ = 1347.38, *p* < 0.001) models. The five factors included challenge stressors, hindrance stressors, creative self-efficacy, the organizational innovation climate, and sustained innovation behavior.

To discuss the between-group difference among employees, we confirmed the cross-level effect before the test. Two MLM layers (Level 1, *N* = 418; Level 2, *N* = 74) were used to estimate the null model. The analysis results revealed significant between-group variance of sustained innovation behavior, revealing that 36% of sustained innovation behavior variance was caused by between-group variance, indicating the suitability of the data for further MLM analysis.

To test the relationship between job stressors and R&D employees’ sustained innovation behavior in H1, MLM was used to test the hypotheses. As shown by Model 2 in Table 3, challenge stressors were found to have significant positive effects on employees’ sustained innovation behavior (γ = 0.28, *p* < 0.01). As a result, H1a was supported. Hindrance stressors were found to have significant negative effects on employees’ sustained innovation behavior (γ = −0.25, *p* < 0.01). As a result, H1b was supported. As shown by Model 4 in Table 3, challenge stressors were found to have significant positive effects on creative self-efficacy (γ = 0.32, *p* < 0.01). As a result, H2b was supported. Hindrance stressors were shown to have significant negative effects on employees’ creative self-efficacy (γ = −0.29, *p* < 0.01). As a result, H2b was supported. In accordance with the mediation effect test reported by Baron and Kenny (1986), Model 5 in Table 3 shows that the coefficients of challenge stressors (γ = 0.24, *p* < 0.01) and hindrance stressors (γ = −0.21, *p* < 0.05) decreased more than those in Model 2, but were still found to be significant. We found that the relationship between creative self-efficacy (γ = 0.28, *p* < 0.01) and sustained innovation behavior was more significant. Creative self-efficacy had a partial mediation effect between job stressors and sustained innovation behavior. Bootstrap analysis was further used for the test. With R software, Monte Carlo analysis was used for extraction 20,000 times, and the maximum likelihood estimation was applied to evaluate 95% confidence intervals (CIs) to determine the significance of indirect effects. The results revealed significant indirect effects (CI between 0.01 and 0.08), supporting H3a and H3b.

**Figure 2:**
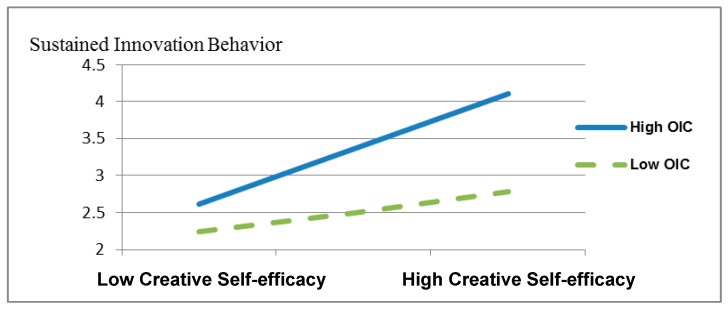
Cross-level moderation effect diagram of the organizational innovation climate (OIC) showing the relationship between creative self-efficacy and sustained innovation behavior.

From Model 6 in Table 3, the χ^2^ test of τ11 was found to be significant, revealing the existence of cross-level moderation effects in the model. In this case, the cross-multiplying terms of creative self-efficacy and the organizational innovation climate were input to form Model 7. In Model 7, with dependent variables of sustained innovation behavior, the results revealed cross-multiplying terms such as 0.29, (t(77) = 5.431, *p* < 0.01), and *R*^2^ = 0.252, showing the cross-level moderation effects of the organizational innovation climate on creative self-efficacy and sustained innovation behavior. Therefore, H4 was supported. To clarify the cross-level moderation effect of the organizational innovation climate, we tested the slope change in the organizational innovation climate to elucidate the relationship between creative self-efficacy and sustained innovation behavior (Figure 2). With a lower organizational innovation climate, creative self-efficacy did not have a notable relationship with sustained innovation behavior (β = 0.08, *p* > 0.050). With a higher organizational innovation climate, creative self-efficacy showed a significant positive relationship with sustained innovation behavior (β = 0.32, *p* < 0.010). When R&D units had a lower organizational innovation climate, the positive relationship between R&D personnel’s creative self-efficacy and sustained innovation behavior decreased.

## 4. Discussion

We discussed the relationships among job stressors, creative self-efficacy, and employees’ sustained innovation behavior and further investigated the role of the organizational innovation climate in sustaining employees’ innovation behaviors. The research design was based on the job demand-resource (JDR) model combined with the social learning theory and expectancy theory. A multi-level analysis was conducted on data gathered from 74 supervisors and 418 employees from 62 technology companies. The research findings indicated the positive effects of challenge stressors on sustaining employees’ innovation behavior, but negative effects of hindrance stressors on sustaining employees’ innovation behavior. Challenge stressors revealed positive effects on employees’ creative self-efficacy, whereas hindrance stressors revealed negative effects on employees’ creative self-efficacy. Creative self-efficacy was found to enhance employees’ sustained innovation behavior and play a mediating role between job stressors and sustained innovation behavior. Finally, the organizational innovation climate was found to affect the relationship between employees’ creative self-efficacy and sustained innovation behavior. In other words, a climate more conducive to organizational innovation or employee confidence in completing innovation tasks facilitate involvement in innovation activity and the occurrence of innovation behavior. The theoretical and practical implications of these findings and the possible research limits and suggestions are summarized below.

### 4.1. Theoretical Contributions

The concept of challenge and hindrance stressors has gradually attracted researchers’ attention and has become another key point in research on stress. The literature supports the idea that regardless of the property difference, challenge stressors have more positive effects on learning motivation, working attitudes, and job performance, whereas hindrance stressors have negative effects [8,9,20,37]. Some studies have indicated that both challenge and hindrance stressors negatively affect employees’ physical and mental health [38,39,40]; however, employees’ physical and mental health directly affect their working behavior. The research results in this study were slightly different from those in previous studies that reported a significant positive relationship between challenge stressors and employees’ sustained innovation behavior, whereas hindrance stressors showed a significant negative relationship with employees’ sustained innovation behavior.

The discussion of the mediation of creative self-efficacy may help researchers in organizations to more carefully explain the cause-effect relationships among variables. We discovered that the role of employees’ creative self-efficacy should be emphasized when discussing the relationship between job stressors and employees’ sustained innovation behavior. This finding reflects the suggestion from organizational innovation research that the work environment in an organization and employees’ sustained innovation behavior should be further discussed [4,19,31]. This finding is also in accordance with Bandura’s [25] efficacy theory about the interactions among individual cognition, situation, and behavior, and the importance of individual initiatives in the situation.

Finally, we applied a multi-level analysis to discuss the effect of the organizational innovation climate on employees’ innovation behavior and the operation mechanism by following Amabile’s [3] research on the organizational innovation climate and responding to the suggestion of Anderson et al. [2]. The results also proved that the organizational innovation climate affect employees’ creative self-efficacy and innovation behavior. The research findings revealed the cross-level moderation effect of the organization level on an individual level, which helps researchers understand the importance of consensus development between organizational leaders and team members. It may assist academia in understanding the multi-level relationships in an organization and help researchers to further comprehend the motivation power in the job characteristics model to promote employees’ creative self-efficacy and innovation behavior.

### 4.2. Implications for Practice

In innovation management practice, the management of employees’ attention poses a challenge for managers because innovation is a complicated and high-risk activity that is full of uncertainties; employees encounter various challenges and bottlenecks in the continuous exploration and attempts to reduce the stagnant state in the innovation process. Managers should adopt positive actions to assist them in dealing with complicated and non-routine work and concentrate on innovation behavior.

The research findings revealed inconsistent effects of challenge and hindrance stressors on individuals. When facing challenge stress, an individual may regard it as motivation to enhance self-development, reach potential, and achieve goals. The development of new products, services, and processes are the major goals of R&D; therefore, challenge stress may enhance innovation behavior. However, excessive stress, e.g., a lack of time and work requirements, may intangibly consume the individual will and dynamics, resulting in physical and mental fatigue and a reduction in work efficiency. Research proved that hindrance stressors, such as workload and supervisor suppression, might negatively affect individual work behavior, e.g., turnover intention, job burnout, and emotional agitation [9,23,24]. Individuals facing the same work pressure might have different responses. In addition, whether stress harms an individual or not depends on individual subjective evaluation. As a consequence, an organization should teach employees to realize that stress is not completely negative by showing that stress can be a motivator to achieve self-development, promoting learning to reinforce self-resilience, learning and searching for solutions to dilemmas and difficulties, or by excluding negative emotions and negative behaviors resulting from stress through the use of organizational resources and communication channels.

We discovered that the organizational innovation climate facilitates employees’ creativity, and creative self-efficacy is the key to ensuring the growth of creativity. In this case, an organization has to implement management measures to enhance members’ innovation behavior and promote organizational innovation performance. An organization, therefore, should create a conducive innovation climate, enhance members’ positive evaluation of the innovation environment, and form organization-level perception sharing to establish members’ sense of innovation security and innovation motivation. We recommend that the human resources management department enhance employees’ sustained innovation behavior from several aspects. First, work could be re-designed to include more autonomous and challenging elements and provide better learning and development opportunities. Second, under the use of teams as the work mode, a good team support environment should be created. The composition of team members can be the key to inducing members’ innovation behavior. For instance, including members who are skilled at creative thinking and innovation practice in a team, or putting members with high and low creative self-efficacy together in innovation programs might facilitate innovation implementation and output. Finally, offering employees annual development reviews can help to identify employees’ future learning needs and can be used to plan the required resources in the organization to help employees reinforce relevant knowledge and skills and strengthen self-work effectiveness through various methods.

### 4.3. Limitations and Future Research

We adopted a cross-sectional study design in which the internal validity of the relationships among job stressors, creative self-efficacy, the organizational innovation climate, and employees sustained innovation behavior was unclear. While different research objects preceded the questionnaire administration, and we discovered the mediation effects of creative self-efficacy on job stressors and employees sustained innovation, as well as the cross-level moderation effects of organizational innovation climate, the cause–effect relationship still requires further verification. We suggest that a longitudinal research design or experimental design should be applied in future studies to analyze the operation of the organizational innovation climate. A questionnaire survey could be used at different time points, or another work design could be used to more clearly understand the effect on employees’ sustained innovation behavior.

The variables in this study were measured with a self-report scale so a same–source bias may have occurred. While employees’ sustained innovation behavior, in this study, was measured according to supervisor evaluation to reduce common method variance, the evaluation might have been affected by the supervisor–subordinate hierarchical connection [41] and other emotional factors. We recommend that other objective work-related variables, e.g., R&D personnel’s actual business achievements and job performance, could be used in future research.

Finally, the generation and practice of creativity is a continuous process. The effects of the organizational innovation climate and creative self-efficacy on individual innovation behavior were proven in this study, but the effects on the generation and practice of creativity were not identified. Factors affecting the enhancement of individual creativity and innovation behavior could be discussed and compared in successive studies to clarify the effect of organizational situations on distinct innovation processes. Empirical research could be conducted to deepen the feedback effect of individual or group creative self-efficacy and should present both theoretical and practical meanings.

## 5. Conclusions

In this study, we used the job demand-resource (JDR) model to discuss the relationships among R&D personnel’s job stressors, creative self-efficacy, the organizational innovation climate, and employees’ sustained innovation behavior. The applicability of job stressors to organizational members’ sustained innovation behavior was successfully analyzed. The mediation effect of creative self-efficacy and the cross-level moderation effect of the organizational innovation climate were identified. Consequently, we found that the job stressors and creative self-efficacy covered in the work conducted in R&D departments of technology companies do not allow R&D personnel to fully develop sustained innovation behavior, but depend on a climate highly conducive to innovation in the organization. Future research should continuously analyze relevant concepts using high working pressure and key predictive factors or moderators of employees’ sustained innovation behavior. We think that that this will enrich the exploration of organizational employees’ sustained innovation behavior and contribute to the advancement of theory and application to practice.

## Figures and Tables

**Table 1:** Mean scores, standard deviations, and correlations among study variables.

Variable	μ	SD	1	2	3	4	5	6
**Individual Level (R&D Employee, *N* = 418)**								
1 Age	34.7	5.33	-					
2 Experience in service	6.8	4.84	0.33 **	-				
3 Challenge stressors	3.31	1.48	0.18 *	0.14	0.91			
4 Hindrance stressors	2.85	1.89	−0.21 *	−0.15	0.31 **	0.88		
5 Creative self-efficacy	3.12	1.44	−0.19 *	−0.18 *	0.25 **	−0.23 **	0.92	
6 Sustained innovation behavior (from supervisor)	3.49	1.97	−0.25 **	−0.17	0.31 **	−0.28 **	0.35 **	0.95
**Firm Level (Supervisor, *N* = 74)**								
Age	41.3	7.46	-					
Experience in service	11.54	3.19	0.22 *	-				
Organizational innovation climate	3.47	1.32	−0.17	−0.15	0.91			

Note: * *p* < 0.05, ** *p* < 0.01; μ, mean; SD, standard deviation.

**Table 2:** Results of discriminative validity by CFA.

Model	χ^2^	df	Δχ^2^	CFI	NFI	RMSEA	SRMR
13-factor model	1347.38	218		0.75	0.82	0.06	0.07
5-factor model	654.73	199	45.27 ***	0.93	0.91	0.05	0.05
Single-factor model	2986.33	231	2342.24 ***	0.51	0.48	0.16	0.12

Note: *** *p* < 0.001; the five-factor model was combined with the organizational innovation climate factors. CFA: Confirmatory Factor Analysis; CFI: Comparative Fit Index; NFI: Normed Fit Index; RMSEA: Root Mean Square Error of Approximation; SRMR: Standardized Root Mean Square Residual.

**Table 3:** Results of the multilevel model (MLM).

	Sustained Innovation Behavior		Creative Self-Efficacy		Sustained Innovation Behavior		
	M1 (SE)	M2 (SE)	M3 (SE)	M4 (SE)	M5 (SE)	M6 (SE)	M7 (SE)
Intercept	4.87 *** (0.081)	4.63 *** (0.537)	4.53 *** (0.511)	4.25 *** (0.513)	4.41 *** (0.557)	4.14 *** (0.474)	4.21 *** (0.496)
Individual Level							
Sex	−0.11 (0.002)	−0.13 (0.004)	−0.09 (0.011)	−0.11 (0.028)	−0.09 (0.019)	−0.05 (0.003)	−0.05 (0.003)
Age	−0.19 * (0.057)	−0.18 (0.079)	0.03 (0.163)	−0.07 (0.151)	−0.15 (0.127)	−0.15 (0.078)	−0.15 (0.078)
Experience in service (R&D employee)	−0.25 * (0.134)	−0.24 * (0.112)	−0.22 * (0.148)	−0.25 * (0.189)	−0.21 * (0.163)	−0.22 * (0.153)	−0.22 * (0.153)
Education	0.22 * (0.082)	0.20 * (0.095)	0.06 (0.124)	0.12 (0.148)	0.19 * (0.144)	0.24 * (0.071)	0.24 * (0.071)
Challenge stressors		0.28 ** (0.076)		0.32 ** (0.227)	0.24 ** (0.136)		
Hindrance stressors		−0.25 ** (0.084)		−0.29 ** (0.194)	−0.21 * (0.107)		
Creative Self-efficacy (CSE)					0.28 ** (0.066)	0.31 ** (0.155)	0.28 ** (0.145)
Firm Level							
Experience in service (supervisor)						0.12 (0.052)	0.08 (0.039)
Organization Innovation Climate (OIC)						0.25 ** (0.061)	0.24 ** (0.046)
OIC × CSE							0.29 ** (0.107)
σ^2^						0.671	0.673
τ_00_						0.503 ***	0.511 ***
τ_11_						0.115 *	0.086 *
*R*^2^ level 1	0.146	0.271	0.137	0.198	0.293	0.128	
*R*^2^ level 2 (maim effect)						0.163	
*R*^2^ level 2 (moderate effect)							0.252
Various of Model							2209.357

Note: * *p* < 0.05, ** *p* < 0.01, *** *p* < 0.001. S.E.: Standard Errors;
